# The influence of hepatitis C infection and interferon-α therapy on thyrotropin blocking and stimulating autoantibodies in Graves' ophthalmopathy: a case report

**DOI:** 10.1186/1756-6614-2-12

**Published:** 2009-12-02

**Authors:** Huy A Tran, Glenn EM Reeves

**Affiliations:** 1 Department of Clinical Chemistry and University of Newcastle, Locked Bag 1, Hunter Region Mail Centre, Newcastle, New South Wales 2310, Australia; 2 Department of Immunopathology and University of Newcastle, Locked Bag 1, Hunter Region Mail Centre, Newcastle, New South Wales 2310, Australia

## Abstract

**Background:**

Hepatitis C virus is a highly immunogenic pathogen often inducing autoimmune activation changes and this can often be further exacerbated by Interferon therapy. As HCV is lymphocytotropic, it can modulate T cell and B cell antibody responses, affecting many endocrine organs, most commonly the thyroid.

**Case presentation:**

We hereby describe a case of fluctuating and wavering thyrotropin autoantibodies of both stimulating and blocking nature in the setting of Graves's ophthalmopathy, hepatitis C infection and interferon-α, causing hypo- and subsequently hyper-thyroidism. The autoantibody profile was clearly modified during interferon therapy and settled into a new equilibrium at the completion of treatment.

**Conclusion:**

The case highlights the possible existence of a dual thyroid autoantibody population associated with hepatitis C, and its modulation by interferon therapy, which further compounds the difficulties in the assessment thyroid disease in this setting.

## Background

Hepatitis C virus is a highly immunogenic pathogen often inducing autoimmune activation changes and this can often be further exacerbated by interferon therapy. As HCV is lymphocytotropic, it can modulate T cell and B cell antibody responses, affecting many endocrine organs, most commonly the thyroid. As a result of this modulating effect, the thyroid autoantibody profile can be severely affected, especially in the setting of Graves' disease superimposed with hepatitis C infection and interferon-based treatment. The following case report illustrates this phenomenon with fluctuating thyrotropin autoantibodies of both stimulating and blocking nature during interferon therapy. It is the existence of these changing antibodies that compounds the difficulties of assessing thyroid disease in this setting.

## Case Presentation

A 44 year-old Caucasian man with chronic hepatitis C infection and known, long-standing primary hypothyroidism presented with recent onset Graves' ophthalmopathy (GO). There was no other medical problem and the patient had otherwise been well. His hypothyroidism was diagnosed 10 years prior, approximately at the same time as his hepatitis C. At that time, the thyrotropin (TSH) was found to be 34 IU/L, free tetra-iodothyronine (fT4) 10.2 and free tri-iodothyronine (fT3) 3.2 pmol/L. He was started on thyroxine therapy with subsequent satisfactory control. No information on autoantibodies or imaging was available from this presentation.

**Figure 1:**
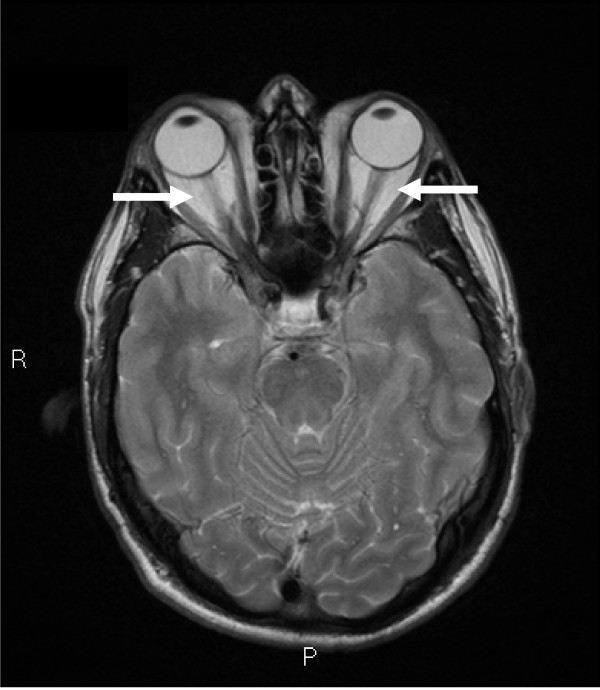
**Magnetic Resonance Imaging of the orbits, showing congestion of the retro-orbital space and enlargement of the extraocular muscles *(arrows)*, consistent with the diagnosis of Graves' Ophthalmopathy**.

Clinical examination showed a tattooed man in euthyroidism. His ophthalmopathy was graded as moderate-to-severe including >3 mm lid retraction and marked congestion with a clinical activity score (CAS) of 5/7 [1]. Visual acuities were 6/6 bilaterally. No goitre, dermatopathy or acanthosis nigricans was detected. His liver span was normal at 11 cm and there was no evidence of chronic liver disease, ascites or portal hypertension. His thyroid ultrasound scan showed 2 small nodules but was otherwise normal in volume. His thyroid pertechnetate uptake scan was reduced at 1% (reference range (RR), 3-8%) whilst on 150 μg of thyroxine daily, at which time his TSH was 1.98 (RR, 0.4-4.0 mU/L) and fT4 21.5 (RR, 10.2-24.5 pmol/L). His antithyroperoxidase and antithyroglobulin antibodies were undetectable. The thyrotropin receptor antibody (TRAb) was 4.0 IU/L (reference interval, < 1.0 IU/L). Other routine laboratory tests were normal including aspartate and alanine aminotransferase activity. His baseline viral load was 6.08 log IU/mL. His ocular magnetic resonance imaging (MRI) supported the diagnosis of GO (Figure 1).

Due to the diametrically opposite clinical findings of hypothyroidism and GO, further bioassays were performed to assess antibodies affecting the function of the TSH receptors. The thyrotropin stimulating antibody (TSAb) was 192 (RR, <180%) and thyrotropin blocking antibody (TBAb) 124 (RR, <40%), suggesting that the mechanism for his hypothyroidism was immune mediated with TSH blocking activity.

### Progress

**Figure 2:**
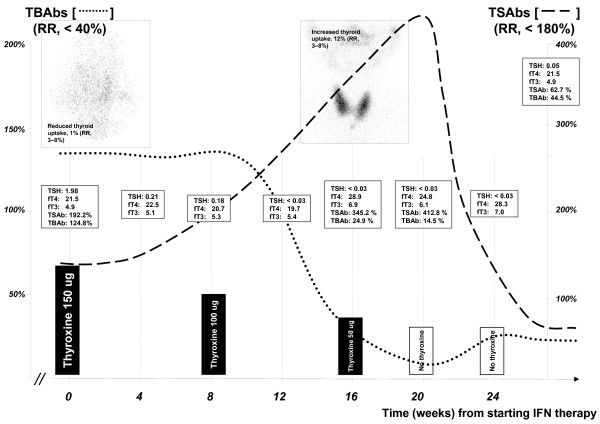
**The immunomodulating effect of Interferon therapy on the TS and TB subsets of autoantibodies and its effects on thyroxine dosage and nuclear uptake patterns**. *Note*: the graphs are only approximate representations, not to exact scale.

Due to the presence of hypothyroidism and the potential additional effect of interferon on thyroid tissue, hepatitis C treatment was carefully started. Because of his hepatitis C genotype 2, liver biopsy was deemed unnecessary and thus was not performed [2]. Treatment then included combination interferon-α and ribavirin for 24 weeks. The ophthalmopathy did not worsen and was managed conservatively with liquid film eye drops and protective glasses. The CAS remained unchanged. Both his eye and thyroid status was monitored and closely reviewed every month. On the 8^th ^week, his TSH declined, necessitating a reduction in thyroxine dosage. At 16 week, he was found to be biochemically thyrotoxic with suppressed TSH, fT4 of 28.9 and fT3 of 6.9 pmol/L at which time his thyroxine was ceased. The TSH stimulating activity increased whilst blocking activity declined. His TSAb rose further whilst blocking activity declined. The evolution of the antibody profile is summarised in Figure 2. The thyroid uptake scan evolved to show a diffuse and increased in uptake at 12%. The potential and aggravating effects of elevated TSAb titres on the ophthalmopathy were duly considered but observation was continued (*see below*).

As the patient was asymptomatic, his thyroid condition was also observed closely. His serial fT4 and fT3 levels remained high but unaltered throughout the course of therapy (Figure 2). Four weeks after the completion of therapy, his TSH was 0.05 mIU/L, fT4 21.5 and fT3 4.9 pmol/L. At 12 weeks, his level had become progressively hypothyroid with TSH of 5.9 mIU/L and fT4 17.9 pmol/L. At the subsequent 6, 12, 24 and 36 months follow-ups, his TSH levels ranged between 5.3 and 6.4 mIU/L. His viral load was undetectable at the 6-month follow-up consistent with sustained virologic response.

## Methods

TRAb assay was measured with the TRAK LUMI test (B.R.A.H.M.S.AG, Hennigsdorf/Berlin, Germany). A TRAb level of <1.0 IU/L is considered negative and > 1.5 as conclusively positive. TSAb and TBAb bioassays were measured as previously described [3,4]. Generally, the detection was carried out in low salt conditioning using JPO9 Chinese hampster ovary cells transfected with the human TSH-R. Cyclic AMP was measured in a commercial RIA (Amersham, Aylesbury, UK). Thyroid stimulation index (SI) was calculated as: SI (percent) = 100 × (cAMP patient/cAMP euthyroid control). For TBAb detection, bovine TSH (1 mU/mL; Sigma, St. Louis, MO) was added with either euthyroid control or test serum. The inhibition index (InI) was calculated as: InI (percent) = 100 × [1 - (counts per minute patient/counts per minute euthyroid control)].

Third generation serum thyrotropin (TSH), serum free tetra- and free tri-iodothyronine (fT4 and fT3) were determined by two-site sandwich immunoassay using an automated chemiluminescent system (Diagnostic Products Corporation, Immulite 2000). The reference range (RR) for TSH was 0.4-4.0 mU/L, fT4 10.0-26.0 and fT3 3.5-5.5 pmol/L. The coefficients of variations (CV) were 5.0% and 5.1% at TSH concentrations of 4.0 mU/L and 10.0 mU/L respectively. For fT4, the CV was 6.5% at 10.0 pmol/L and fT3 8.9% at 3.5 pmol/L.

Serum autoantibodies to thyroglobulin and thyroperoxidase were measured by agglutination (Serodia-ATG and Serodia-AMC, Fujirebio, Inc., Tokyo, Japan). Titres of less than 1:400 were considered normal for both.

## Discussion

Although the co-existence of dual thyroid TSAb and TBAb has been well documented [5], its pattern in the presence of hepatitis C and its modulation by interferon treatment has rarely been reported previously [6]. The present case highlights the population of thyroid autoantibodies whose evolution is significantly influenced in the presence of hepatitis C and interferon. It was highly likely that the initial diagnosis of hypothyroidism was the result of TSH blocking activity resulting in the biochemical expression of hypothyroidism. In the absence of further information at the time of diagnosis, it was hard to confirm this hypothesis. However, in favour of this diagnosis was the absent/negligible nuclear uptake in the thyroid scan, the presence of a normal-size thyroid on ultrasound and the absence of any thyroid auto-antibodies. Although excessive thyroxine can affect thyroid uptake scan appearance, in the presence of a normal (non-suppressed) TSH level, it is very likely that the uptake scan reflects the activity of TBAbs.

The development of GO is a fascinating feature that must closely involve the presence of TRAb. Although the pathogenesis of GO remains undetermined, TSAb is one of the major contributors in inducing the inflammatory process in the orbital fat and ocular muscles resulting in swelling and congestion of the orbit [7]. In addition, recent case reports suggested an association between hepatitis C infection and GO [8,9]. Hypothetically, prior to the development of GO, there must have existed an equilibrium between these two sub-classes of thyroid antibodies, albeit unbalanced in favour of TBAb, just sufficient to elicit the hypothyroidism but with enough stimulating activity to participate in the development of GO. In the presence of interferon as an immuno-modulator, the equilibrium then shifted in favour of TSAb and hence the progressive thyrotoxic biochemical profile with marked nuclear uptake. It was conceivable but unlikely that this shift was spontaneous and coincidental because his thyroid status was stable in the intervals following interferon therapy. This would have been further bolstered if his thyroid parameters were available in the period preceding hepatitis C treatment. These changes appeared permanent as the hypothyroidism moderated compared to before treatment. Despite the mildly elevated TSH level, no thyroxine was required 3 years after the completion of interferon therapy. This is underlined by the newly equilibrated antibody profile at the end of treatment, with both TS- and TB-Ab subclass activities being normal. These were not performed further in the convalescing period. Interestingly, the relatively higher TS-Ab activity did not further compound the ophthalmopathy.

The management of GO in this case was both challenging and difficult, involving plentiful of discussion with the patient and his spouse. As mentioned, the addition of interferon may aggravate the GO which in turn may be further exacerbated by the evolving TSAb, potentially precipitating an ophthalmic crisis and loss of vision [9]. On the other hand, independent treatment with immunosuppressants such as glucocorticoid, calcineurin inhibiting agents or methotrexate can potentially lead to fulminant hepatic necrosis and failure. After much deliberation and risk estimation, the GO was monitored closely with visual acuities and color charts weekly, tapering to monthly as the condition remained progressively stable. Although the natural history of this condition is unknown in this setting and to be safe, orbital irradiation and decompressive surgery were also consulted and made readily available. Fortunately, his ophthalmic condition did not deteriorate.

The underlying pathogenesis of this swinging antibody profile is unknown. Although the prevalence of hypothyroidism in the setting of hepatitis C and interferon is not uncommon, this rare type of hypothyroidism often goes unsuspected unless there are other indicators such as ophthalmopathy. The antibodies switching to the TSH receptors must be modulated, evolving from blocking to predominantly stimulating. It is both a relief and fascination that GO failed to progress as TSAb has been suggested to initiate and stimulate orbital adipogenesis [10]. Recent studies were able to further investigate the inhibitory and stimulatory nature of these antibodies. In fact monoclonal antibodies with both stimulating and block activities were recently developed. Plausibly the relevant B cells are selected to alter the variable regions of the immunoglobulins to specifically affect the critical areas for TSH receptors stimulation and blockade, particularly region M22 and 5C9 of the TSH autoantibodies [11,12]. It must be noted however that the switching between hyper- and hypothyroid phases of Graves' disease can occur independent of hepatitis C infection and interferon therapy [13]

In HCV infection, there is also an increased secretion of IFN-γ and chemokine ligand 10 (CXCL10) generally as well as by thyrocytes [14]. The high CXCL10 level is higher in patients who develop thyroid disease, especially hypothyroidism in the setting of hepatitis C. It is possible that the endo-, exo-genous interferon and CXCL 10 combine to modify the immune response. The elevated CXCL10 level has also been implicated in GO recently [15]. These factors may add to the aforementioned mechanisms to result in the complex immuno-chemical cascade representative of this case. This is purely speculative however and this observed immune phenomenon remains poorly understood. It adds to the spectrum of thyroid diseases in the presence of hepatitis C and in particular its modulation by interferon therapy [16].

## Conclusion

This case highlights another fascinating and complex interaction between the thyroid and interferon therapy in hepatitis C infection. Although highly unusual, hypothyroidism potentially due to TBAb should be thoroughly considered in this setting.

## Consent

Written informed consent was obtained from the patient for publication of this case report and any accompanying images. A copy of the written consent is available for review by the Editor-in-Chief.

## Competing interests

The authors declare that they have no competing interests.

## Authors' contributions

Both authors contribute equally and substantially to, and approved the final version of the manuscript.
